# ^68^Ga-Labeled GX1 Dimer: A Novel Probe for PET/Cerenkov Imaging Targeting Gastric Cancer

**DOI:** 10.3389/fonc.2021.750376

**Published:** 2021-09-30

**Authors:** Jipeng Yin, Bo Xin, Mingru Zhang, Xiaoli Hui, Na Chai, Hao Hu, Bing Xu, Jing Wang, Yongzhan Nie, Guangqing Zhou, Guanliang Wang, Hongbing Lu, Liping Yao, Liusheng Chen, Kaichun Wu

**Affiliations:** ^1^ School of Biomedical Engineering, Fourth Military Medical University, Xi’an, China; ^2^ Clinical Medical Research Center, The 75th Group Army Hospital of Chinese People’s Liberation Army (PLA), Dali, China; ^3^ Department of Oncology, No. 960 Hospital of PLA, Taian, China; ^4^ Department of Nuclear Medicine, Xijing Hospital, Fourth Military Medical University, Xi’an, China; ^5^ First Affiliated Hospital, Xi’an Jiaotong University, Xi’an, China; ^6^ State Key Laboratory of Cancer Biology, National Clinical Research Center for Digestive Diseases and Xijing Hospital of Digestive Diseases, Fourth Military Medical University, Xi’an, China

**Keywords:** gastric cancer, GX1 dimer, PET imaging, Cerenkov imaging, TGM2

## Abstract

**Purpose:**

To synthesize the dimer of GX1 and identify whether its affinity and targeting are better than those of GX1. To prepare ^68^Ga-DOTA-KEK-(GX1)_2_ and to apply it to PET and Cerenkov imaging of gastric cancer.

**Methods:**

^68^Ga-DOTA-KEK-(GX1)_2_ was prepared, and the labeling yield and stability were determined. Its specificity and affinity were verified using an *in vitro* cell binding assay and competitive inhibition test, cell immunofluorescence, and cell uptake and efflux study. Its tumor-targeting ability was determined by nano PET/CT and Cerenkov imaging, standardized uptake value (SUV), signal-to-background ratio (SBR) quantification, and a biodistribution study in tumor-bearing nude mice.

**Results:**

^68^Ga-DOTA-KEK-(GX1)_2_ was successfully prepared, and the labeling yield was more than 97%. It existed stably for 90 min in serum. The binding of ^68^Ga-DOTA-KEK-(GX1)_2_ to cocultured HUVECs (Co-HUVECs) was higher than that to human umbilical vein endothelial cells (HUVECs), BGC823 cells, and GES cells. It was also higher than that of ^68^Ga-DOTA-GX1, indicating that the dimer did improve the specificity and affinity of GX1. The binding of KEK-(GX1)_2_ to Co-HUVECs was significantly higher than that of GX1. Additionally, the uptake of ^68^Ga-DOTA-KEK-(GX1)_2_ by Co-HUVECs was higher than that of ^68^Ga-DOTA-GX1 and reached a maximum at 60 min. Nano PET/CT and Cerenkov imaging showed that the tumor imaging of the nude mice injected with ^68^Ga-DOTA-KEK-(GX1)_2_ was clear, and the SUV and SBR value of the tumor sites were significantly higher than those of the nude mice injected with ^68^Ga-DOTA-GX1, indicating that the probe had better targeting *in vivo*. Finally, the biodistribution showed quantitatively that when organs such as the kidney and liver metabolized rapidly, the radioactivity of the tumor site of the nude mice injected with ^68^Ga-DOTA-KEK-(GX1)_2_ decreased relatively slowly. At the same time, the percentage of injected dose per gram (%ID/g) of the tumor site was higher than that of other normal organs except the liver and kidney at 60 min, which indicated that the tumor had good absorption of the probe.

**Conclusion:**

GX1 was modified successfully, and the *in vivo* and *in vitro* properties of the GX1 dimer were significantly better than those of GX1. The imaging probe, ^68^Ga-DOTA-KEK-(GX1)_2_, was successfully prepared, which provides a candidate probe for PET and Cerenkov diagnosis of gastric cancer.

## Introduction

Gastric cancer is one of the malignant tumors with the highest morbidity and mortality (1). The survival time of patients with gastric cancer has been prolonged after decades of conquest. However, most patients are diagnosed in the middle and late stages, so they cannot be cured surgically. Early diagnosis is the key to reducing the mortality of gastric cancer. But traditional diagnosis methods, including gastroscope and barium x-ray examination, cannot meet the needs of early diagnosis. Therefore, a new method has been widely sought.

Molecular imaging is getting more and more attention, and it can show early changes in tumors at the molecular level, especially with the application of nuclear medicine (2). Functional imaging can be achieved, and radionuclides can be used for treatment at the same time (3). The key to molecular imaging is to screen molecules that can target the target cells or tissues and make the targeted molecules into imaging probes or radiotherapy drugs, which can be used in the diagnosis and internal radiotherapy of gastric cancer.

Tumor vascular targeting imaging and treatment have become increasingly mature (4). The tumor vascular targeting peptide GX1 was screened in a gastric cancer-transplanted tumor mouse model using a phage peptide library (5). In the early stage, GX1 was used as a target molecule to obtain a large amount of experimental data, such as ^99m^Tc-GX1 (an imaging probe) (6, 7) and GX1-rmhTNFα (a fusion protein) (8, 9), which can be used in the imaging and treatment of gastric cancer. However, as a short peptide, GX1 also has inherent shortcomings, such as insufficient affinity for the receptor, short circulation time *in vivo*, and weak targeting ability. Therefore, the modification of GX1 is the focus of our research.

In this paper, ^68^Ga-DOTA-KEK-(GX1)_2_, a novel probe, was prepared using GX1 peptide. The specificity and affinity of the new dimer were verified by cell immunofluorescence, *in vitro* cell receptor binding assay, cell uptake and efflux assay. Nano PET/CT imaging, Cerenkov imaging, and biodistribution were used to verify its targeting effect *in vivo*.

It must be proven that the dimer of GX1 is better than the monomer before GX1 can be further developed and applied. At the same time, the nuclide ^68^Ga is popular in current studies and has been gradually incorporated into clinical applications (10). Therefore, in this paper, not only was GX1 modified to achieve better results, but ^68^Ga was also developed and applied to provide new ideas for the diagnosis of gastric cancer.

## Materials and Methods

### Cell Culture and Animal Model

The coculture system was used to simulate human umbilical vein endothelial cells (HUVECs) *in vitro*. The cells were routinely cultured in M200 medium. HUVECs and the human gastric adenocarcinoma cell line BGC823 in logarithmic growth phase were digested, centrifuged, resuspended, and inoculated into the upper and lower layers of a Transwell petri dish (10 cm) with a aperture of 0.4mm. In the subsequent cell experiment, the conditioned medium was added to the pore plate to maintain the stimulation state of the coculture, and the conditioned medium was the culture supernatant of BGC823 cells, which was filtered and diluted with M200 culture medium at 1:4.

All of the experiments were performed according to a protocol approved by the Fourth Military Medical University (FMMU) Animal Care and Use Committee. A total of 0.2 ml of cell suspension (BGC823, 2 × 10^6^ cells) was implanted by subcutaneous injection into the right upper limb of athymic female nude BALB/c mice (age, 4–6 weeks; weight, 15–20 g). When the diameter of the tumor reached approximately 8 mm, it could be used for imaging.

### Synthesis and Isotope Labeling of Peptides

**Figure 1:**
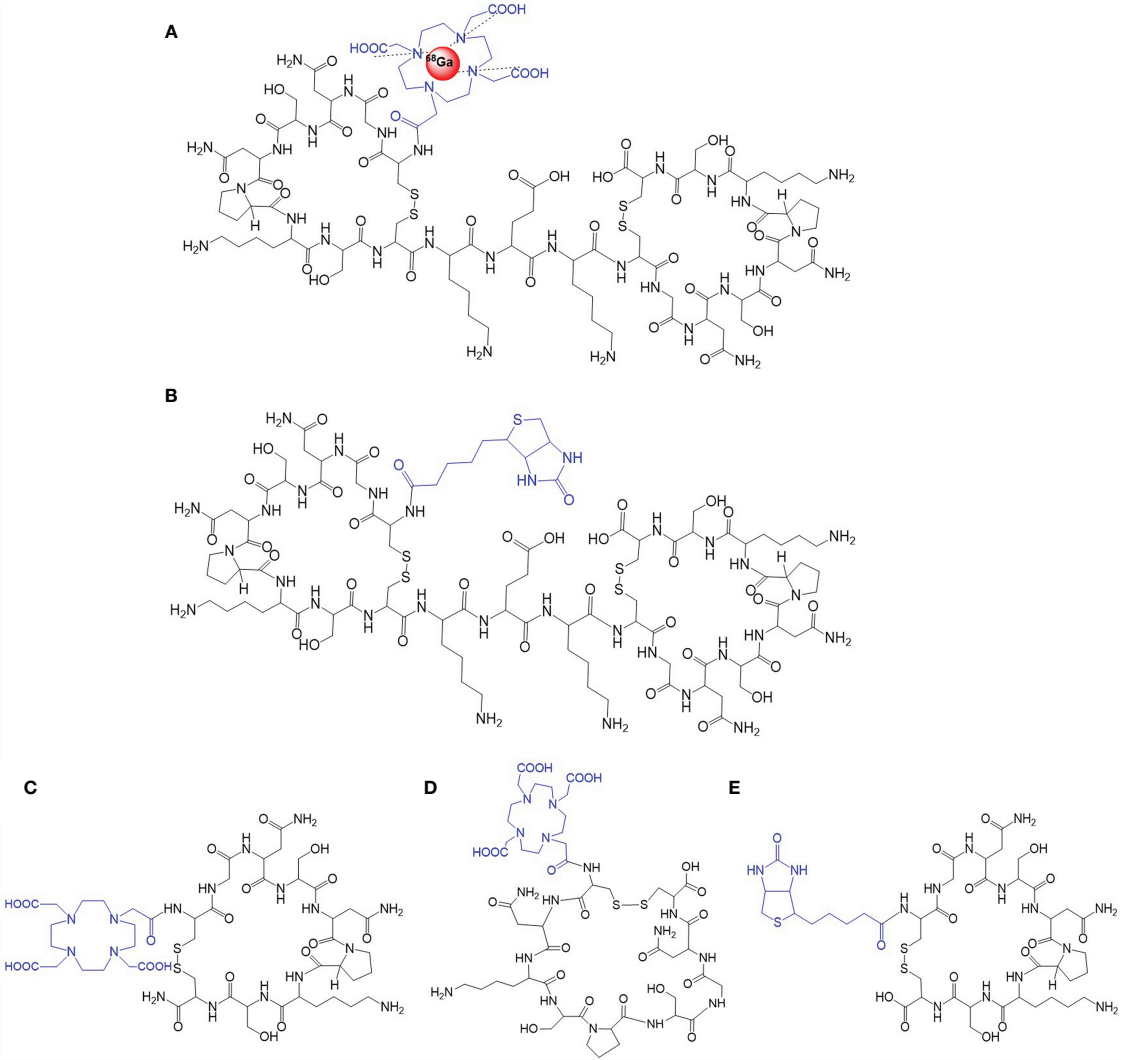
The structure of the probes. **(A)** ^68^Ga-DOTA-KEK-(GX1)_2_ **. (B)** Biotin-KEK-(GX1)_2_. **(C)** DOTA-GX1. **(D)** DOTA-URP. **(E)** Biotin-GX1.

All peptides were synthesized by GL Biochem (Shanghai, China), including Biotin-GX1, Biotin-KEK-(GX1)_2_, DOTA-GX1, DOTA-URP, and DOTA-KEK-(GX1)_2_ (**Figure 1**). They were stored at -20°C after freeze-drying. The purity was identified by high-performance liquid chromatography (HPLC), and the molecular weight was identified by mass spectrometry.

^68^Ga-DOTA-GX1 was prepared using a one-step method. In brief, 10 μg of DOTA-GX1, 50 μl of sodium acetate buffer (1.25 mol/L), and 1 ml of ^68^GaCl_3_ were added to the reaction system. Then, it was boiled for 10 min. The labeling rate was determined, and the bacteria were removed by a 0.22-μm filter for subsequent cell and animal experiments.

### *In Vitro* Stability Analysis

We used four kinds of solutions to observe the stability of the probes, namely, raw solution, normal saline, fresh mouse serum, and human serum. The specific methods were as follows: the probe and each solution were mixed according to a volume ratio of 1:100, and the original solution was kept as a control. The radiochemical purity of the solution was determined at 0, 30, 60, and 90 min. A curve was drawn to observe the stability of the probe *in vitro* and to speculate whether the probe would be removed from the standard *in vivo*.

### Immunofluorescence Staining

The binding of GX1 and KEK-(GX1)_2_ on Co-HUVECs was observed by immunofluorescence. The cells were implanted into the immunofluorescence cell culture chamber, fixed for 10 min using ice-cold acetone after the cells were adhered and fused to 70%, and incubated with 1% bovine serum albumin–phosphate buffered saline (BSA-PBS) for 30 min. Biotin-GX1 or Biotin-KEK-(GX1)_2_ (0.01 mg/ml) was added to the cell chambers at 4°C overnight. Fluorescein isothiocyanate (FITC)-streptavidin (Abcam, Cambridge, MA, USA) was added at 1:300 for 1 h with 4′,6-diamidino-2-phenylindole (DAPI; 25 ng/ml; Roche, Mannheim, Germany) for 15 min. The cells were observed using a confocal fluorescence microscope (FV10i, Olympus, Tokyo, Japan).

### *In Vitro* Radioligand Binding Assay and Receptor Competitive Inhibition Assay

This experiment was used to observe the binding of ^68^Ga-DOTA-KEK-(GX1)_2_ to different cells, such as Co-HUVECs, HUVECs, BGC 823 cells, and an immortalized human gastric mucosal epithelial cell line (GES). There was only one kind of probe, which was divided into four groups according to the cells. The cells were added to 48-well plates (3 × 10^4^/well). Each cell group was divided into three study groups, namely, the experimental group, competitive group, and control group, and each study group comprised three wells. BSA (1%) was added into each well for 30 min. Then, ^68^Ga-DOTA-KEK-(GX1)_2_ (3.7 × 10^5^ Bq) was added to the experimental group, ^68^Ga-DOTA-KEK-(GX1)_2_ and 25 mmol/L unlabeled GX1 were added to the competitive group, and PBS without the probe was added to the control group. The orifice plate was placed for 30 min at 4°C. The cells were digested and collected, and the liquid reading in each well was measured by a gamma counter.

This experiment was also used to observe the binding of ^68^Ga-DOTA-KEK-(GX1)_2_, ^68^Ga-DOTA-GX1, and ^68^Ga-DOTA-URP to Co-HUVECs. There was only one kind of cell, which was divided into three groups according to the probes. Each probe was divided into three groups, namely, the experimental group, competitive, group and control group, following the same steps described before.

### Cell Uptake and Efflux

For cell uptake, Co-HUVECs were plated into 24-well plates (10^5^/well), with three wells as a group. Then, ^68^Ga-DOTA-KEK-(GX1)_2_, ^68^Ga-DOTA-GX1, or ^68^Ga-DOTA-URP was added at an activity of 1.85 × 10^5^ Bq. The cells were split and collected using NaOH (0.1 mol/L) after incubation for 0, 30, 60, and 90 min. The cell lysate was measured by a gamma counter. For the efflux study, the cells were incubated with the probes for 90 min at 37°C. Then, the cells were split and collected, following the same steps as before.

### Nano PET/CT Imaging

Nano PET/CT imaging of tumor-bearing nude mice was used to observe the targeting of the probes against gastric cancer and whether they could be blocked by GX1. A total of 3.7 × 10^6^ Bq of probe was injected into the mice *via* the tail vein, with three mice in each group. A 10-minute static scan was acquired at 30, 60, or 90 min after injection under 2% isoflurane-maintained anesthesia using Nano PET/CT (Mediso, Hungary). The images were reconstructed by a two-dimensional ordered-subsets exception maximum algorithm. The regions of interest (ROIs) were circled after imaging to calculate the standardized uptake value (SUV) of the tumor site.

### Cerenkov Imaging

A total of 18.5 × 10^6^ Bq of probe was injected into each mouse *via* the tail vein. The Cerenkov images were recorded using an IVIS Lumina II spectrum imaging system. The images were acquired at 30, 60, and 90 min under isoflurane anesthesia (exposure time: 60 s, f/stop = 8, binning = 1). The ROIs were drawn over the tumor, and the signal-to-background ratio (SBR) was calculated.

### Biodistribution

In this experiment, the biodistribution of three probes and the biodistribution of ^68^Ga-DOTA-KEK-(GX1)_2_ at different time points in tumor-bearing nude mice were observed. For the biodistribution of the probes, 3.7 × 10^6^ Bq of probe was injected intravenously. The nude mice were sacrificed under euthanasia, and the organs were excised, weighed, and counted at 60 min. The percentage of injected dose per gram (%ID/g) was calculated. For the biodistribution of ^68^Ga-DOTA-KEK-(GX1)_2_ at different time points, the mice were sacrificed after injection at 30, 60, and 90 min. The %ID/g was calculated as described previously.

### Statistical Analysis

All data are expressed as the means ± SD. Statistical analysis was performed by one-way analysis of variance (ANOVA) using IBM SPSS Statistics. The level of significance was set at p < 0.05.

## Results

### Successful Labeling and Stability

**Figure 2:**
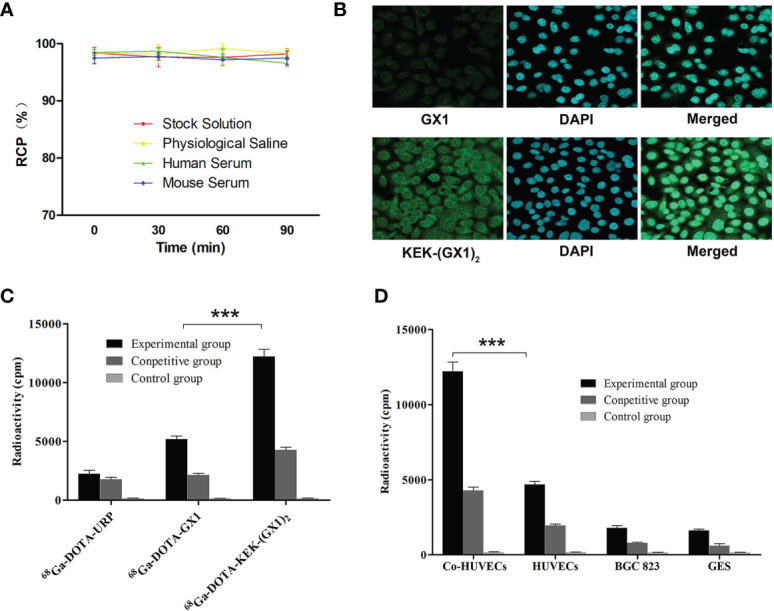
**(A)** The stability of ^68^Ga-DOTA-KEK-(GX1)_2_. There was no significant reduction in radiochemical purity (RCP) in each solution, and it was speculated that ^68^Ga-DOTA-KEK-(GX1)_2_ was stable *in vitro* and *in vivo.* **(B)** Cell immunofluorescence analysis of KEK-(GX1)_2_ expression in Co-HUVECs (×200). Co-HUVECs were incubated with KEK-(GX1)_2_, GX1, and 4′,6-diamidino-2-phenylindole (DAPI). In addition, the peptides and DAPI were merged. The results indicate that the binding ability of KEK-(GX1)_2_ is much higher than that of GX1. **(C, D)** Receptor binding assay and competitive inhibition assay with various cell types incubated with ^68^Ga-DOTA-KEK-(GX1)_2_, ^68^Ga-DOTA-GX1, or ^68^Ga-DOTA-URP in the absence or presence of GX1. The binding of ^68^Ga-DOTA-KEK-(GX1)_2_ to Co-HUVECs was higher than that of ^68^Ga-DOTA-KEK-(GX1)_2_ to HUVECs (***p < 0.001) and was also higher than that of ^68^Ga-DOTA-GX1 to Co-HUVECs (***p < 0.001).

The labeling yield measured by radioactive thin-layer chromatography (TLC) was more than 97%. Four kinds of solutions were used to observe the stability of the probe, namely, raw solution, normal saline, fresh mouse serum, and human serum. The probe was mixed with each solution at a volume ratio of 1:100, and the original solution was retained as a control. The radiochemical purity of the solution was determined after incubation for 0, 30, 60, and 90 min at 37°C. As shown in **Figure 2A**, the labeled peptide stably existed in various solutions for 90 min, and the labeling rate remained above 95%. It is speculated that the labeled peptide will not be delabeled *in vivo*.

### Cell Immunofluorescence

Immunofluorescence staining was used to observe whether GX1 and KEK-(GX1)_2_ can bind to Co-HUVECs and which peptide has higher binding ability. As shown in **Figure 2B**, both GX1 and KEK-(GX1)_2_ could bind to Co-HUVECs at the same concentration, but the binding ability of KEK-(GX1)_2_ was much higher than that of GX1.

### Receptor Binding Affinity and Specificity

^68^Ga-DOTA-KEK-(GX1)_2_ can specifically bind to Co-HUVECs, as shown in **Figure 2D**. The binding of ^68^Ga-DOTA-KEK-(GX1)_2_ to Co-HUVECs was higher than that of HUVECs (p < 0.001) and much higher than that of BGC 823 and GES. At the same time, it was blocked by unlabeled GX1.

^68^Ga-DOTA-KEK-(GX1)_2_ has a stronger binding ability to Co-HUVECs. As shown in **Figure 2C**, the binding of ^68^Ga-DOTA-KEK-(GX1)_2_ to Co-HUVECs was higher than that of ^68^Ga-DOTA-GX1 (p < 0.001) and much higher than that of ^68^Ga-DOTA-URP (p < 0.001). At the same time, it was blocked by unlabeled GX1. The results showed that compared with ^68^Ga-DOTA-GX1, the binding ability of ^68^Ga-DOTA-KEK-(GX1)_2_ to the receptor was greatly enhanced.

### Cell Uptake and Efflux

**Figure 3:**
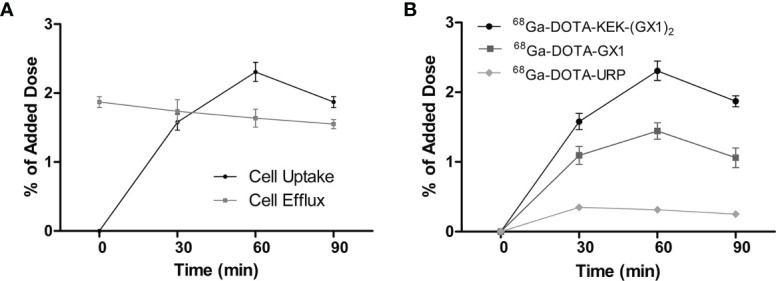
**(A)** Cell uptake and efflux studies of ^68^Ga-DOTA-KEK-(GX1)_2_ in Co-HUVECs. **(B)** Cell uptake of ^68^Ga-DOTA-KEK-(GX1)_2_, ^68^Ga-DOTA-GX1, and ^68^Ga-DOTA-URP in Co-HUVECs. The background readings are reflected at 0 min.

As shown in **Figure 3A**, the uptake of ^68^Ga-DOTA-KEK-(GX1)_2_ by Co-HUVECs increased before 60 min, reached a maximum at 60 min, and then decreased. However, the efflux from 1 to 90 min decreased slowly. The efflux was higher than the uptake at 0–30 min, and the uptake from 60 to 90 min was higher than the efflux. Therefore, the difference value between uptake and efflux was the largest over 60 min, and it can be inferred that the best effect may be achieved in 60 min during *in vivo* imaging. Meanwhile, the uptake of ^68^Ga-DOTA-KEK-(GX1)_2_ and ^68^Ga-DOTA-GX1 by Co-HUVECs was always higher than that of ^68^Ga-DOTA-URP within 90 min and reached the maximum at 60 min. However, the uptake of ^68^Ga-DOTA-KEK-(GX1)_2_ by Co-HUVECs was higher than that of ^68^Ga-DOTA-GX1 (p < 0.05) (**Figure 3B**).

### ^68^Ga-DOTA-KEK-(GX1)_2_ Has Good Targeting of Gastric Cancer *In Vivo*

**Figure 4:**
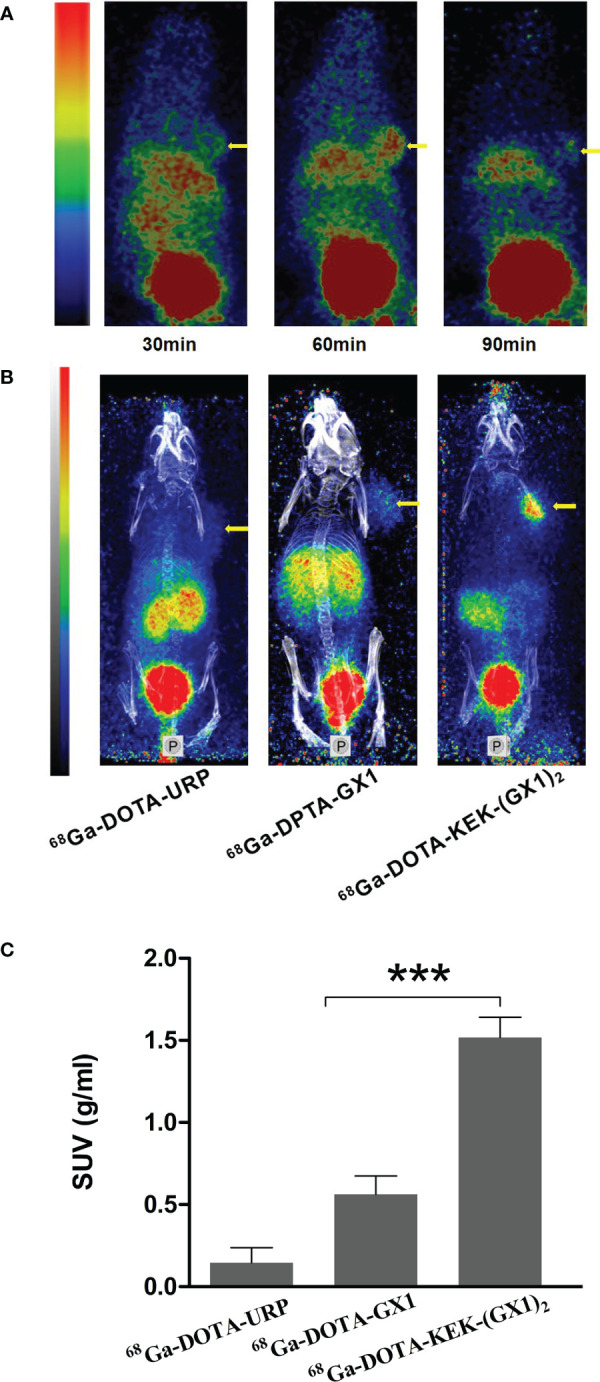
**(A)** Nano PET/CT imaging of subcutaneous BGC-823 tumor-bearing nude mice at 30, 60, and 90 min after intravenous injection of 3.7 × 10^6^ Bq of ^68^Ga-DOTA-KEK-(GX1)_2_. **(B)** Nano PET/CT imaging of tumor-bearing nude mice at 60 min postinjection of equal radioactive ^68^Ga-DOTA-KEK-(GX1)_2_, ^68^Ga-DOTA-GX1, and ^68^Ga-DOTA-URP. The tumors are shown as yellow arrows. **(C)** The standardized uptake value (SUV) of the tumor site. The SUV of the ^68^Ga-DOTA-KEK-(GX1)_2_ group was higher than that of the ^68^Ga-DOTA-GX1 group (n = 3/group, ***p < 0.001).

**Figure 5:**
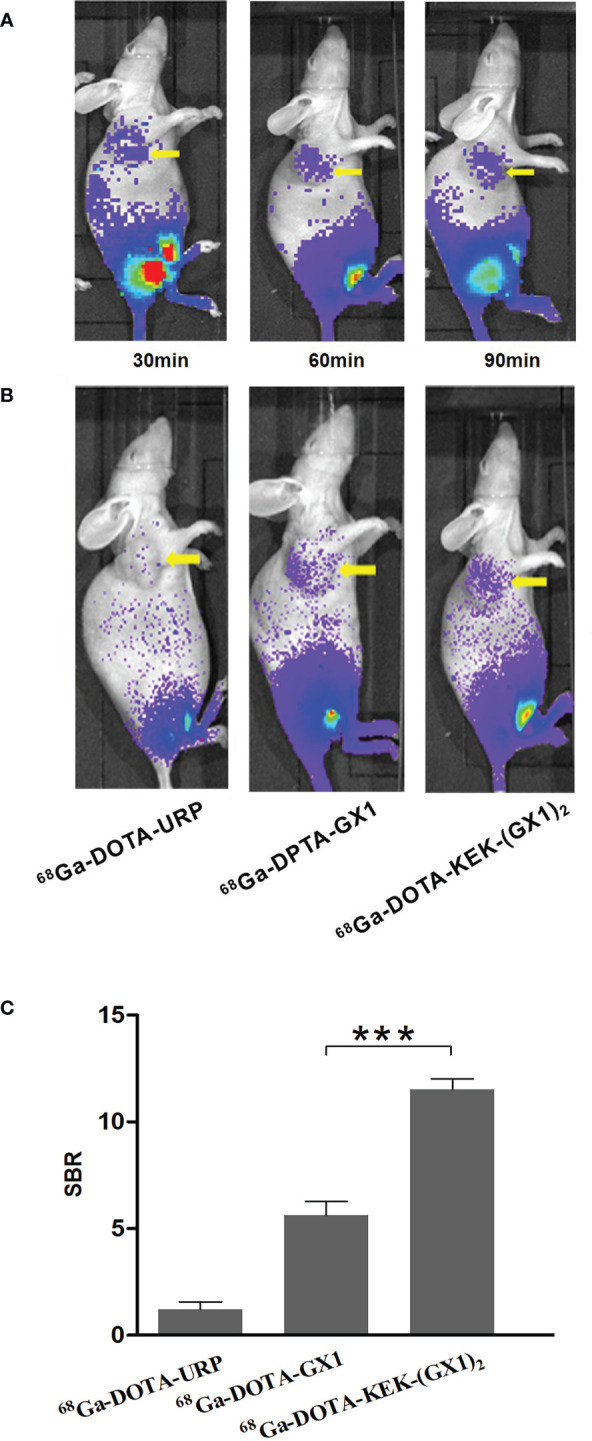
**(A)** Cerenkov imaging of subcutaneous BGC-823 tumor-bearing nude mice at 30, 60, and 90 min after intravenous injection of 3.7 × 10^6^ Bq of ^68^Ga-DOTA-KEK-(GX1)_2_. **(B)** Nano PET/CT imaging of tumor-bearing nude mice at 60 min postinjection of equal radioactive ^68^Ga-DOTA-KEK-(GX1)_2_, ^68^Ga-DOTA-GX1, and ^68^Ga-DOTA-URP. The tumors are shown as yellow arrows. **(C)** The signal-to-background ratio (SBR) of the tumor site. The SBR of the ^68^Ga-DOTA-KEK-(GX1)_2_ group was higher than that of the ^68^Ga-DOTA-GX1 group (n = 3/group, ***p < 0.001).

Tumor-bearing nude mice were divided into three groups and injected with ^68^Ga-DOTA-GX1, ^68^Ga-DOTA-URP, or ^68^Ga-DOTA-KEK-(GX1)_2_. The injection dose was 3.7 × 10^6^ Bq, and a 10-min static scan was acquired at 30, 60, or 90 min. The results are shown in **Figures 4A, B**. The results of nano PET/CT imaging confirmed that there was better uptake of ^68^Ga-DOTA-KEK-(GX1)_2_ at the tumor site over 60 min, and the dimer had better tumor targeting than that of the monomers. In addition, to quantitatively observe the distribution of the probe, the SUV of the tumor site was calculated (**Figure 4C**). The SUV of the ^68^Ga-DOTA-KEK-(GX1)_2_ group was higher than that of the ^68^Ga-DOTA-GX1 group (p < 0.001), indicating that the tumor uptake of ^68^Ga-DOTA-KEK-(GX1)_2_ was significantly increased. Similar results were obtained by Cerenkov imaging (**Figure 5**).

### Biodistribution Study

**Figure 6:**
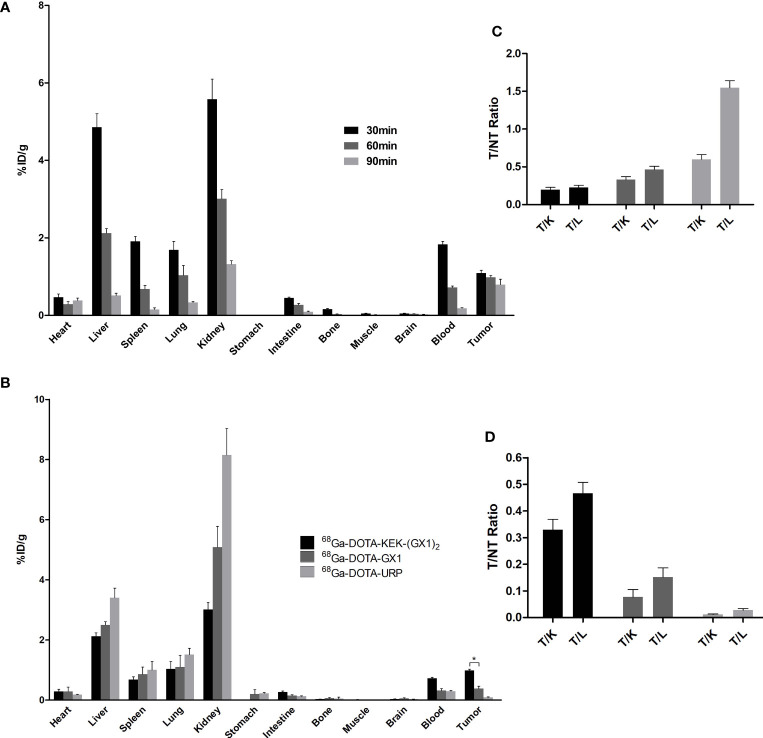
**(A)** Biodistribution of nude mice bearing BGC-823 tumors injected with ^68^Ga-DOTA-KEK-(GX1)_2_. The T/NT ratios are shown in panel **(C)** All of the ratios increased over time, illustrating that ^68^Ga-DOTA-KEK-(GX1)_2_ had the ability to specifically target tumors *in vivo*. **(B)** Biodistribution of nude mice bearing BGC-823 tumors at 60 min postinjection of equal radioactive ^68^Ga-DOTA-KEK-(GX1)_2_, ^68^Ga-DOTA-GX1, and ^68^Ga-DOTA-URP. In addition, T/NT ratios were shown in panel **(D)** The uptake of ^68^Ga-DOTA-KEK-(GX1)_2_ by tumors was much higher than that of ^68^Ga-DOTA-GX1 (*p < 0.05). T, tumor; NT, nontumor; K, kidney; L, liver.

In this experiment, the distribution of ^68^Ga-DOTA-KEK-(GX1)_2_ in tumor-bearing nude mice was observed and divided into 30-, 60-, and 90-min groups. The nude mice were sacrificed in batches after injection at different times. The organs were taken, weighed, and counted, and the %ID/g was calculated. As shown in **Figures 6A, C**, when radioactivity of the kidney and liver reduced rapidly, the radiation of the tumor decreased relatively slowly. At the same time, at 60 min, except for the liver and kidney, the %ID/g of the tumor was higher than that of other normal organs, which indicated that the tumor had better absorption of the probe.

Additionally, the distribution of ^68^Ga-DOTA-KEK-(GX1)_2_, ^68^Ga-DOTA-GX1, and ^68^Ga-DOTA-URP in tumor-bearing nude mice was observed. The nude mice were sacrificed at 60 min; the organs were taken, weighed, and counted; and the %ID/g was calculated. As shown in **Figures 6B, D**, the absorption of the three probes was highest in the kidney, followed by the liver, indicating that they were mainly metabolized by the kidney and liver and excluded from the body. The absorption of ^68^Ga-DOTA-KEK-(GX1)_2_ and ^68^Ga-DOTA-GX1 by tumors was higher than that of ^68^Ga-DOTA-URP, and the uptake of ^68^Ga-DOTA-KEK-(GX1)_2_ by tumors was much higher than that of ^68^Ga-DOTA-GX1 (p < 0.05).

## Discussion

In a previous article (7), we introduced the advantages and disadvantages of polyethylene glycol (PEG) modification. To avoid the influence of PEG modification, we prepared a new dimer, KEK-(GX1)_2_, to determine whether the dimer has better affinity and targeting than the monomer. This dimer is linked by glutamic acid and lysine, which is a common method currently. A large number of studies support this modification (11, 12). After the peptide was synthesized successfully, ^68^Ga-DOTA-KEK-(GX1)_2_ was prepared by coupling the chelating agent DOTA and ^68^Ga labeling, and its properties were identified *in vitro* and *in vivo*. The probe was characterized by labeling yield and stability and then confirmed by cell immunofluorescence. The binding of KEK-(GX1)_2_ to Co-HUVECs was significantly higher than that of monomer GX1, which was consistent with the results of cell receptor binding analysis *in vitro*. The results of cell receptor binding analysis and competitive inhibition tests *in vitro* showed that the binding of ^68^Ga-DOTA-KEK-(GX1)_2_ to Co-HUVECs was higher than that of HUVECs, BGC823 cells, and GES cells, indicating that the probe specifically bound to tumor vascular endothelial cells. The experimental results also showed that the binding of ^68^Ga-DOTA-KEK-(GX1)_2_ to Co-HUVECs was higher than that of ^68^Ga-DOTA-GX1, indicating that the dimer did improve the affinity of GX1. The cell uptake and efflux experiments showed that the uptake of ^68^Ga-DOTA-KEK-(GX1)_2_ by Co-HUVECs was higher than that of ^68^Ga-DOTA-GX1 and reached the maximum at 60 min. Nano PET/CT imaging results showed that the tumor imaging of nude mice injected with ^68^Ga-DOTA-KEK-(GX1)_2_ was clear, and the SUV of the tumor site was significantly higher than that of nude mice injected with ^68^Ga-DOTA-GX1, indicating that the probe has better targeting *in vivo*. Finally, we used the biodistribution to quantitatively observe the distribution of the probe in the tumor-bearing nude mice. The results showed that when organs such as the kidney and liver metabolized rapidly, the radioactivity of the tumor site of the nude mice injected with ^68^Ga-DOTA-KEK-(GX1)_2_ decreased relatively slowly, and at the same time, the %ID/g of the tumor site was higher than that of other normal organs except the liver and kidney at 60 min, which indicated that the tumor had good absorption of the probe.

Importantly, we used URP as the control throughout the experiment, and we also prepared ^68^Ga-DOTA-URP. Through a comparative study, we prepared a new type of probe that can be used for the diagnosis of gastric cancer by PET imaging. The probe is a dimer of GX1, which makes up for the lack of affinity of GX1 and has good application prospects.

The nano PET/CT used in this study is essentially different from the single photon emission computed tomography (SPECT) used previously. In this part of the study, we used nano PET/CT with small animal imaging equipment, and the resolution and sensitivity were greatly improved (13). In addition, PET and SPECT, collectively known as emission computed tomography (ECT), are the most sensitive molecular imaging modes (14). In addition, imaging is not limited by tissue depth. Nuclear medicine imaging is widely carried out worldwide because of its many advantages (15). In terms of detection efficiency, the highest efficiency of SPECT is only 1%–3% that of PET, and the quality of SPECT image acquisition is not as good as the diagnostic efficiency of PET. Therefore, the application of PET is representative of the highest level of development of nuclear medicine (16). The nuclides used in PET examination (such as ^18^F, ^11^C, ^13^N, ^15^O, etc.) generally have a short half-life and are ultrashort half-life nuclides, and the irradiation time to patients is short. Although there are many advantages, PET also has disadvantages. Most of the nuclides used in PET are produced by cyclotrons, and the equipment cost is high (17). Therefore, the application of PET is limited. The study of nuclides with convenient sources is one of the directions for the development of PET. In this study, we chose ^68^Ga. ^68^Ga is obtained by leaching from the ^68^Ge-^68^Ga generator (18). Its source is convenient, and its half-life is short, only 68 min. The generator half-life is 288 days, and it can be used for a long time, even more than 1 year. The development of a ^68^Ge-^68^Ga generator has also undergone a long-term process. At present, the best method is to use a generator to adsorb ^68^Ge on a SnO_2_ column, elute with ultrapure hydrochloric acid to obtain ^68^GaCl_3_, and then label other molecules in the form of a complex (19). Because the half-life is short, the labeling method needs to save time. Through the exploration of conditions, we have established a set of methods for labeling short peptides with DOTA, which is simple to operate and requires a short amount of time (20–22). However, our research also had shortcomings, including very high blood uptake. It may be due to the fact that it takes several hours or even longer for the peptide to achieve maximum binding to the receptor in the circulation *in vivo*. And the half-life of ^68^Ga is too short, thus the imaging time can only be controlled within 90 min. Therefore, there are still many free probes in the circulation of plasma. Furthermore, from cell immunofluorescence staining, the PEG-(GX1)_2_ could enter into cells. But we do not know how it gets inside of cells. Now, the receptor of GX1 has been identified, that is, TGM2. Interaction between GX1 and TGM2 will help elucidate the mechanism of GX1 internalization.

The morbidity and mortality of gastric cancer are very high, and the situation is not optimistic in East Asia, especially in China (23). Thus, it is necessary to study gastric cancer. The low early detection rate of gastric cancer is one of the main reasons for the high mortality rate of gastric cancer (24, 25). Finding a new method for the diagnosis of gastric cancer is key to conquering gastric cancer. The imaging of gastric cancer using tumor vascular-targeted peptides and molecular imaging technology is an important method for the early diagnosis of gastric cancer (26, 27). We sought to use GX1 with independent intellectual property rights to image gastric cancer (28–31). However, previous studies found that the targeting and affinity of GX1 still need to be improved, and there is still some distance to go before application. The main purpose of this study was to modify GX1 and then prepare an effective probe that can better image gastric cancer. Therefore, in this part of the study, the dimer of GX1 [KEK-(GX1)_2_] was prepared from glutamic acid and lysine and then coupled with the chelating agent DOTA and labeled with ^68^Ga to prepare ^68^Ga-DOTA-KEK-(GX1)_2_. It was found that the characteristics of the probe were better than those of monomer GX1 *in vivo* and *in vitro*.

## Conclusion

In summary, we prepared a dimer of GX1, which has higher gastric cancer targeting and affinity. The labeling method of 68Ga, a new radionuclide in the clinic, was explored, which laid the foundation for the further development and application of GX1 and ^68^Ga.

## Data Availability Statement

The original contributions presented in the study are included in the article/supplementary material. Further inquiries can be directed to the corresponding authors.

## Author Contributions

JY, BXi, and MZ contributed equally. LY and KW designed the study. JY, BXi, MZ, XH, NC, HH, and BXu performed the experiments. JY, BXi, and LC analyzed the data and wrote the manuscript. MZ and JW evaluated the images. YN, GZ, GW, HL, and KW contributed reagents and materials. All authors contributed to the article and approved the submitted version.

## Conflict of Interest

The authors declare that the research was conducted in the absence of any commercial or financial relationships that could be construed as a potential conflict of interest.

## Publisher’s Note

All claims expressed in this article are solely those of the authors and do not necessarily represent those of their affiliated organizations, or those of the publisher, the editors and the reviewers. Any product that may be evaluated in this article, or claim that may be made by its manufacturer, is not guaranteed or endorsed by the publisher.
